# Global-genome Nucleotide Excision Repair Controlled by Ubiquitin/Sumo Modifiers

**DOI:** 10.3389/fgene.2016.00068

**Published:** 2016-04-28

**Authors:** Peter Rüthemann, Chiara Balbo Pogliano, Hanspeter Naegeli

**Affiliations:** Institute of Pharmacology and Toxicology, Vetsuisse Faculty, University of ZurichZurich, Switzerland

**Keywords:** aging, cyclobutane pyrimidine dimer, DNA repair, genomic instability, photoproducts, sunburns, skin cancer, UV radiation

## Abstract

Global-genome nucleotide excision repair (GG-NER) prevents genome instability by excising a wide range of different DNA base adducts and crosslinks induced by chemical carcinogens, ultraviolet (UV) light or intracellular side products of metabolism. As a versatile damage sensor, xeroderma pigmentosum group C (XPC) protein initiates this generic defense reaction by locating the damage and recruiting the subunits of a large lesion demarcation complex that, in turn, triggers the excision of aberrant DNA by endonucleases. In the very special case of a DNA repair response to UV radiation, the function of this XPC initiator is tightly controlled by the dual action of cullin-type CRL4^DDB2^ and sumo-targeted RNF111 ubiquitin ligases. This twofold protein ubiquitination system promotes GG-NER reactions by spatially and temporally regulating the interaction of XPC protein with damaged DNA across the nucleosome landscape of chromatin. In the absence of either CRL4^DDB2^ or RNF111, the DNA excision repair of UV lesions is inefficient, indicating that these two ubiquitin ligases play a critical role in mitigating the adverse biological effects of UV light in the exposed skin.

## Introduction

All organisms are constantly under attack by environmental and endogenous DNA-damaging agents that endanger the sequence fidelity of their genomes. Many environmental mutagens cause “bulky” DNA adducts that destabilize the complementary pairing of bases in the native double helix (Straub et al., 1977; Knox et al., 1987). Base pair-destabilizing lesions also result from internal by-products of cellular metabolism including oxygen radicals (Brooks et al., 2000; Kuraoka et al., 2000), but the most common type of bulky DNA lesion arises from the UV spectrum of sunlight or indoor tanning devices, generating covalent crosslinks joining neighboring pyrimidines, i.e., CPDs and pyrimidine-pyrimidone (6-4) photoproducts (6-4PPs; Brash, 1988). If not readily repaired, these pyrimidine crosslinks and other bulky adducts interfere with transcription, DNA replication or cell cycle progression (Lopes et al., 2006; Brueckner et al., 2007), eventually giving rise to mutations and chromosomal aberrations that accelerate aging and culminate in cancer (Marteijn et al., 2014). Unfortunately, the incidence of skin cancer continues to increase and remains a public health concern despite widespread knowledge that excessive exposure to sunlight is the major risk factor for cutaneous neoplasms (Donaldson and Coldiron, 2011; Usher-Smith et al., 2014). This review is focused on recent advances in our knowledge of how polypeptide modifiers regulate the DNA repair response preventing sunlight-induced skin cancer.

### Excision of Bulky DNA Lesions

Nucleotide excision repair is a molecular cut-and-patch machine that removes bulky base lesions by incising damaged DNA strands on either side of the injury, thereby eliminating 24- to 32-nucleotide long single-stranded segments (Huang et al., 1992; Moggs et al., 1996). Depending on their location in the genome, bulky lesions are sensed by two alternative mechanisms. The TC-NER pathway is initiated when an RNA polymerase II complex encounters obstructing base lesions (Bohr et al., 1985). Such transcriptional roadblocks trigger a stepwise reaction for the rapid removal of base lesions from transcribed strands (reviewed by Hanawalt and Spivak, 2008; Vermeulen and Fousteri, 2013; Marteijn et al., 2014). On the other hand, GG-NER activity is generally slower but detects bulky lesions anywhere in the genome independently of transcription (reviewed by Scharer, 2013; Puumalainen et al., 2016). Genetic defects in the GG-NER pathway cause XP, which is a severe cancer-prone syndrome presenting with photosensitivity, extreme sunburns and an over 1,000-fold higher risk of contracting sunlight-induced neoplasms of the skin (Hollander et al., 2005; DiGiovanna and Kraemer, 2012). Patients suffering from the XP syndrome are classified into distinct genetic complementation groups (from XP-A to XP-G) reflecting mutations in respective NER genes (Cleaver et al., 2009). A variant form of this disease (XP-V) is caused by mutations in a gene coding for DNA polymerase η that catalyzes with high nucleotide sequence fidelity the replicative bypass of UV lesions in S phase of the cell division cycle (Masutani et al., 1999).

The initial detection of bulky lesions in the GG-NER pathway is carried out by a three-subunit factor consisting of XP group C protein (XPC; Sugasawa et al., 1998; Volker et al., 2001) one of two human RAD23 homologs (predominantly RAD23B; Ng et al., 2003) and (CETN2, (Araki et al., 2001; Nishi et al., 2005; Dantas et al., 2011). The DNA-binding activity of this heterotrimeric complex resides with the XPC subunit itself. RAD23B and CETN2 contribute by supporting the proper folding of XPC protein and by protecting this DNA-binding subunit from degradation (Ng et al., 2003; Xie et al., 2004; Krasikova et al., 2012). Although RAD23B stimulates the recognition of damaged DNA by XPC protein (Sugasawa et al., 1996), it is readily released once XPC associates with DNA lesion sites (Fei et al., 2011; Bergink et al., 2012). Conversely, CETN2 remains associated with target sites (Dantas et al., 2013) where XPC provides a platform for the recruitment of TFIIH. This 10-subunit complex contains an ATPase (XPB) and a DNA helicase (XPD) that separate complementary strands to produce an unwound configuration of about 25 nucleotides around the lesion (Evans et al., 1997; Wakasugi and Sancar, 1998). Stability to the resulting open intermediate or “bubble” is conferred by XPA together with RPA, until the DNA strand containing the damage is incised by structure-specific endonucleases exactly at the double-stranded to single-stranded DNA transitions on each side of the bubble (Evans et al., 1997; Missura et al., 2001; Li et al., 2015). A protein heterodimer composed of XPF and ERCC1 introduces the incision on the 5′ side, followed by incision on the 3′ side by the endonuclease activity of XPG (Staresincic et al., 2009). After this dual incision and consequent release of the excised oligonucleotide carrying the damage, the remaining single-stranded gap is filled by DNA repair synthesis by the action of DNA polymerases η, 𝜀, or κ (Ogi et al., 2010). Ligation by DNA ligase I and DNA ligase IIIα finally restores helix integrity (Araujo et al., 2000; Moser et al., 2007).

### Structure and Interactome of the XPC Initiator

**FIGURE 1:**
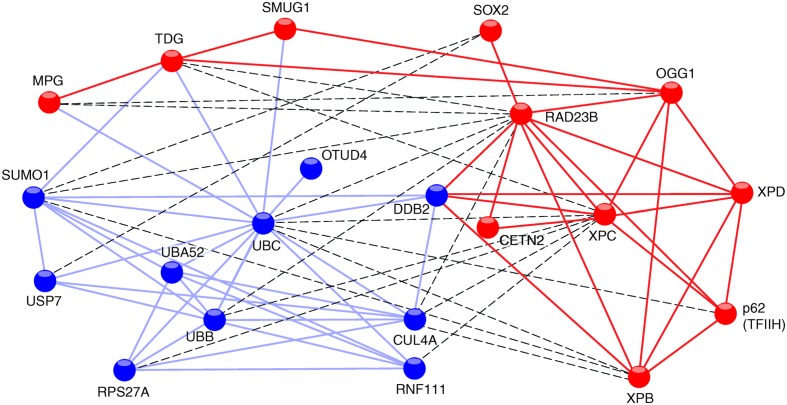
**STRING network view of XPC interactions with proteins.** The connecting lines indicate proven or predicted interactions using the http://www.string-db.org information source. The different colors of the protein nodes reflect their clustering in two groups according to the KMEANS algorithm (Brohée and Helden, 2006). Blue nodes, ubiquitin-related proteins; red nodes, DNA repair proteins. Blue lines, interactions between ubiquitin-related proteins; red lines, interactions between DNA repair proteins. The dashed lines highlight interactions between the two different clusters.

The human XPC polypeptide is made of 940 amino acids and harbors domains for binding to DNA (Hey et al., 2002; Yasuda et al., 2005; Trego and Turchi, 2006) and multiple protein partners (**Figure 1**). Its molecular structure can be extrapolated from that of Rad4 protein, the evolutionarily conserved homolog in the yeast *Saccharomyces cerevisiae* (Min and Pavletich, 2007). When undergoing co-crystals with a model bulky lesion in duplex DNA, Rad4 protein deploys four adjacent domains for substrate binding by two different modalities. One part makes use of a TG domain and a BHD1, which cooperate in associating with 11 base pairs of duplex DNA flanking the damaged site. The second part uses two further BHD2 and BHD3 to interact with four consecutive nucleotides of the undamaged DNA strand opposing the flipped-out bulky lesion. No interactions at all are formed with the lesion itself. In human XPC protein, this β-hairpin region (BHD1–3) interacting indirectly with damaged sites encompasses amino acids 637–831 (Camenisch et al., 2009).

In addition to mediating associations with substrate DNA, the TG domain is required for the interaction between Rad4 and Rad23 (Min and Pavletich, 2007), and between the corresponding human homologs XPC and RAD23B. A fraction of the human TG domain also interacts with XPA protein (Bunick et al., 2006). Another partner, known as DDB2 does not exist in lower eukaryotes like yeast. However, a transient association between DDB2 and XPC is critical for the processing of CPDs in mammals (Itoh et al., 2004) and the respective contact sites have been mapped to the TG and BHD1 regions (Fei et al., 2011). Residues 847–863 in the carboxy-terminus of human XPC form an α-helix that binds tightly to CETN2 (Nishi et al., 2005; Yang et al., 2006). Amino acid residues 816–940 located in this carboxy-terminus and a portion of the amino-terminal region around amino acid position 334 make contacts with two members (p62 and XPB) of the 10-subunit TFIIH complex (Yokoi et al., 2000; Uchida et al., 2002; Bernardes de Jesus et al., 2008). These particular interactions reflect the actual role of XPC in recruiting the XPD helicase, another TFIIH subunit, which in turn detects lesions by scanning DNA and sequestering damaged nucleotides in a dedicated recognition pocket on its enzyme surface (Sugasawa et al., 2009; Mathieu et al., 2010). In addition, XPC protein interacts with the following base excision repair enzymes: MPG, (Miao et al., 2000), TDG, (Shimizu et al., 2003), OGG1, (D’Errico et al., 2007; Melis et al., 2011), and SMUG1, (Shimizu et al., 2010). This crosstalk with multiple DNA glycosylases indicates that XPC may adopt a more general function in recruiting diverse repair enzymes to base pair-disrupted sites in the double helix. Perhaps the most unexpected interaction of XPC protein is with the Oct4-Sox2 transcriptional activator. Indeed, the XPC complex was found to serve as a coactivator of the Oct4-Sox2-dependent expression of the Nanog pluripotency gene (Fong et al., 2011; Cattoglio et al., 2015; Zhang et al., 2015). A two-hybrid screen, which used XPC protein as the bait, revealed many further potential interaction partners involved in DNA synthesis, transcription, post-translational modification, proteolysis, signal transduction, and metabolism (Lubin et al., 2014). To date, the biological consequence of these putative associations is unknown. Finally, there are also proven interactions of XPC protein with two different deubiquitinases, i.e., OTUD4, (Lubin et al., 2014) and USP7 deubiquitinase (for *U*biquitin-*S*pecific-processing *P*rotease 7; He et al., 2014). It appears, therefore, that XPC upon ubiquitination becomes a substrate for these two deubiquitinating enzymes.

### Support for the XPC Initiator from a Specialized UV Lesion Detector

Exposure of DNA to UV light results in the formation of CPDs and 6-4PPs in a stoichiometry of approximately 3:1. These two kinds of pyrimidine crosslinks differ in their biophysical properties, genomic distribution, and biological effects. First, CPD sites are characterized by a relatively minor destabilization of base pairs compared to duplex DNA containing 6-4PPs (Kim et al., 1995; Jing et al., 1998; McAteer et al., 1998). Second, CPDs are evenly distributed across the chromatin landscape, whereas 6-4PPs are formed preferentially in linker DNA segments rather than in nucleosome cores (Gale et al., 1987; Gale and Smerdon, 1990; Mitchell et al., 1990). Third, because CPDs are removed at slower rates than 6-4PPs, they display a higher mutagenic potential and are responsible for most adverse short- and long-term effects of UV radiation such as sunburns, skin aging and cutaneous cancer (Schul et al., 2002; Garinis et al., 2005).

Despite being the generic repair initiator for all bulky lesions including the slowly repaired CPDs, XPC protein does not bind CPDs in duplex DNA with any appreciable selectivity (Sugasawa et al., 2001; Hey et al., 2002; Reardon and Sancar, 2003; Wittschieben et al., 2005). This lack of specificity for CPDs is, however, compensated by DDB2 protein, which is the factor mutated in XP-E patients (Nichols et al., 2000; Kulaksiz et al., 2005). Unlike XPC, which functions as a non-specific sensor of helix-disrupting bulky lesions, DDB2 is exclusively dedicated to the detection of CPDs and 6-4PPs (Tang et al., 2000). Structural analyses of DDB2 crystals revealed a recognition hole in its central β-propeller fold that only accommodates CPDs and 6-4PPs while excluding larger base adducts (Scrima et al., 2008; Fischer et al., 2011; Yeh et al., 2012; Osakabe et al., 2015). Notably, the complete lack of functional DDB2 protein in XP-E patients abolishes the repair of CPDs but the excision of 6-4PPs is only marginally affected (Hwang et al., 1999; Moser et al., 2005).

A generally proposed model is that DDB2 recognizes CPDs and, thereafter, delivers them to the XPC partner for initiation and execution of the GG-NER process (Tang et al., 2000; Wakasugi et al., 2001; Fitch et al., 2003). It has been demonstrated that XPC lends two of its previously mentioned DNA-binding folds (TG domain and BHD1) to interact in a transient manner with DDB2 associating with UV lesions. This dynamic DDB2-XPC-DNA intermediate at the damage site allows for the insertion, into the DNA double helix, of a β-hairpin extension protruding from BHD3, eventually competing DDB2 away from the damage (Fei et al., 2011; Mu et al., 2015). Thermodynamically, this β-hairpin insertion by XPC takes place at a considerable energetic cost for local breakage of stacking and hydrogen bond interactions between the involved bases (Mu et al., 2015). The 6-4PPs, being more base pair-disruptive, facilitate this β-hairpin insertion by reducing the helical stability at damaged sites, but XPC protein depends on DDB2 to interact in a productive manner with CPD sites. Thus, the different degree of local helical distortion explains the specific defect of XP-E cells in eliminating CPD lesions.

### Polypeptide Modifiers Targeting XPC Protein

In view of the manifold implications of XPC as a generic DNA quality sensor in GG-NER that, in addition, associates with several DNA glycosylases and is responsible for non-repair functions in transcription (see above), it is not astonishing to observe that the activity, cellular level and localization of XPC protein is tightly controlled. For example, it has become clear that various polypeptide modifiers regulate the action of this versatile repair initiator during the cellular response to UV damage.

In addition to its role as a specific UV lesion detector, the DDB2 subunit cooperates with the adaptor DDB1 to recruit the CUL4A scaffold and the RING finger protein ROC1, which together build the CRL4^DDB2^ ubiquitin ligase. By mediating the covalent attachment of one or more 8-kDa ubiquitin moieties to target proteins (Groisman et al., 2003), this cullin-type ligase is able to fine-tune GG-NER activity. Under steady-state conditions, the CRL4^DDB2^ ubiquitin ligase is kept in an inactive form thanks to an association with the COP9 signalosome, a multi-subunit regulatory protease (Fischer et al., 2011). Following the detection of UV lesions by DDB2, COP9 is released giving way to a covalent modification of CUL4A with the ubiquitin-like polypeptide NEDD8, thus activating the ubiquitin ligase complex that, in turn modifies nearby located substrates with Lys48-linked ubiquitin chains (Scrima et al., 2008). The principal ubiquitination substrates include histones H2A, H3 and H4 as well as DDB2 itself and its DNA recognition partner XPC (Nag et al., 2001; Sugasawa et al., 2005; Kapetanaki et al., 2006; Wang et al., 2006; Guerrero-Santoro et al., 2008).

It has been proposed that the CRL4^DDB2^-mediated ubiquitination of histones in response to UV radiation helps opening chromatin, thus facilitating access of the GG-NER repair machinery to damaged DNA (Wang et al., 2006). However, this view is contradicted by the finding that CUL4A conditional-knockout mice show more proficient rather than reduced GG-NER activity (Liu et al., 2009). There is, on the other hand, general agreement that the self-ubiquitination of DDB2 not only suppresses its binding to DNA but also promotes its degradation by the 26S proteasome (Sugasawa et al., 2005). The same CRL4^DDB2^ ligase also ubiquitinates XPC but, unlike the fate of DDB2, XPC retains its DNA-binding property and is shielded from proteasomal breakdown (Sugasawa et al., 2005; Matsumoto et al., 2015). In addition, the XPC protein is modified with Lys63-linked ubiquitin chains by another ligase complex referred to as RNF111 or Arkadia (Poulsen et al., 2013). This extra ubiquitination reaction is strictly dependent on the prior UV-dependent modification of XPC protein with sumo, defining RNF111 as a sumo-targeted ubiquitin ligase (Wang et al., 2005).

In summary, GG-NER activity upon UV damage is coordinated by several polypeptide modifiers including NEDD8, sumo, Lys48- and Lys63-linked ubiquitin chains. Sumo and the two aforementioned ubiquitin chains decorate XPC protein at multiple covalent modification sites. Interestingly, *in situ* immunofluorescence studies indicate that a down-regulation of CRL4^DDB2^ or RNF111 activity has opposite effects by inhibiting and stimulating, respectively, the accumulation of XPC in damage spots generated by UV irradiation through micropore filters. This observation raises the possibility that Lys48-linked ubiquitin chains (produced by CRL4^DDB2^) and Lys63-linked counterparts (produced by RNF111) have distinct modulating roles. The function of Lys48-linked ubiquitin chains in regulating XPC is discussed in the next section below. With regard to the accompanying sumo modification, this reaction has been implicated in promoting the release of DDB2 once XPC is bound to UV lesion sites. In the absence of XPC sumoylation, both DDB2 and XPC are trapped together on damaged DNA carrying the lesion, thus posing a block to downstream NER steps (Akita et al., 2015). Since RNF111 is targeted to protein substrates by sumo residues, it is tempting to propose that the effect of sumoylation in releasing XPC may actually be executed by a subsequent attachment of Lys63-linked ubiquitin chains by RNF111. This functional link between sumo and Lys63-linked ubiquitin would explain the persistence of XPC in UV lesion spots observed by Poulsen et al. (2013) and van Cuijk et al. (2015) following RNF111 depletion.

### Dynamic Relocation of XPC in Damaged Chromatin

The genome packaging in eukaryotic cells is imposed by two very diverging needs. The DNA filaments must be compressed to fit into the narrow cellular nucleus but nevertheless remain accessible to the diverse nuclear transactions. To achieve this double requirement, DNA is assembled with histones to generate a tight but dynamic array whose repeating unit is the nucleosome (reviewed by Khorasanizadeh, 2004; Thoma, 2005). Each individual nucleosome displays a core particle, where 147 base pairs of duplex DNA are wrapped around a core histone octamer (two each of H2A, H2B, H3, and H4) and a DNA spacer or “linker” of variable length. Also, in higher eukaryotes histone H1 associates with linker DNA segments to induce further packaging allowing for increased compaction of the DNA double helix.

It is of paramount importance to address the possible regulatory role of polypeptide modifiers in the GG-NER pathway taking into account this chromatin context. New insights into the function of CRL4^DDB2^-mediated ubiquitination came from the enzymatic partitioning of chromatin by incubation with micrococcal nuclease (MNase). This particular enzyme breaks down DNA in the more accessible linker segments much faster than in the less accessible nucleosome cores. As a consequence, the incubation of chromatin with MNase produces a soluble supernatant of mostly non-histone proteins that, before MNase digestion, were associated with linker DNA segments spacing the nucleosomal core particles (amounting to ∼35% of total genomic DNA). Even when saturating enzyme concentrations are used, however, MNase digestions of chromatin leave behind the vast majority of nucleosome core particles (amounting to ∼60% of total DNA) in the form of an insoluble nucleoprotein fraction (Telford and Stewart, 1989). Two previous findings led us to predict that, in response to UV irradiation, CRL4^DDB2^ activity would not be uniformly distributed along nucleosome arrays. First, DDB2 protein, the DNA-binding subunit of CRL4^DDB2^, associates with > 10-fold higher affinity with 6-4PPs (*K*_a_ = 1.5 × 10^9^ M^-1^) relative to CPDs (*K*_a_ = 1 × 10^8^ M^-1^; (Reardon et al., 1993; Wittschieben et al., 2005). Second, 6-4PPs are formed mainly in internucleosomal linker DNA (Gale and Smerdon, 1990; Mitchell et al., 1990). Therefore, we were not surprised to find that DDB2 associates preferentially, although not exclusively, with 6-4PPs situated in accessible MNase-sensitive internucleosomal segments (Fei et al., 2011). Coversely, it was believed that XPC is unable to interact with DNA assembled with histone octamers forming nucleosome cores (Yasuda et al., 2005) but, against this prevailing notion, MNase digestions of chromatin revealed that XPC protein associates rather evenly with nucleosome core particles and internucleosomal linker segments. Upon UV irradiation, this interaction of XPC protein with nucleosome core particles is stimulated (Fei et al., 2011). This latter finding is in line with structural analyses of core particle crystals containing a site-directed UV damage, which revealed that the tight wrapping around histone octamers increases the DNA flexibility at lesion sites (Osakabe et al., 2015). This higher flexibility may, in turn, explain how XPC protein is able to carry out, even in the nucleosome core context, its indirect damage sensor function by binding to the undamaged strand opposing bulky lesions.

**FIGURE 2:**
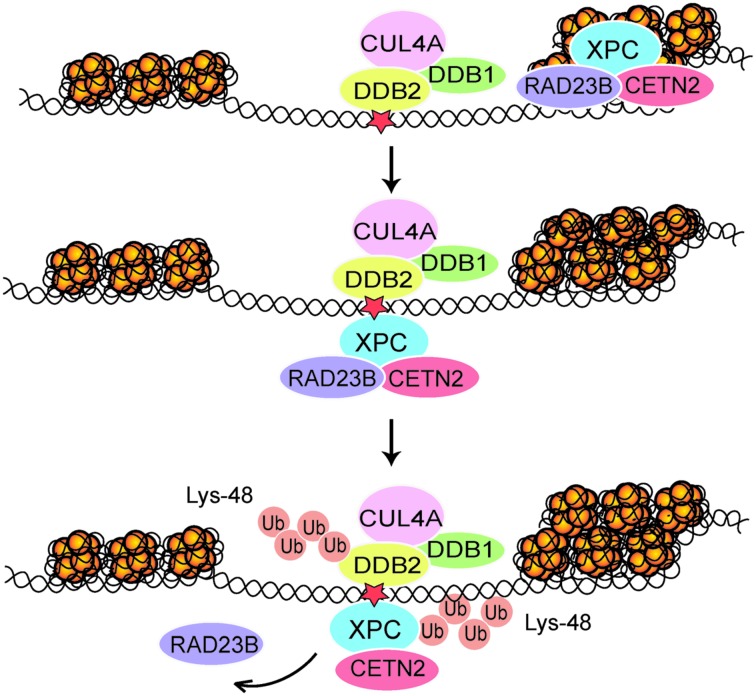
**Regulation of XPC localization in chromatin.** After each UV pulse, the cullin-type CRL4^DDB2^ ligase complex (comprising *inter alia* DDB1, DDB2, and CUL4A) is recruited mostly to accessible internucleosomal sites in chromatin. The ensuing modification of XPC with Lys48-linked ubiquitin (Ub) chains leads to a temporary retention of XPC on internucleosomal DNA, thus reducing its constitutive association with nucleosome core particles (Fei et al., 2011). Subsequently, RAD23B is released and the XPC-CETN2 heterodimer provides a platform for recruitment of the TFIIH complex. The UV radiation damage is symbolized by a red star.

In agreement with the selectivity of the DDB2 subunit for UV lesions in internucleosomal linker DNA, following UV radiation the whole CRL4^DDB2^ ubiquitin ligase is relocated mainly to these highly amenable sites. Due to this distinctive positioning of CRL4^DDB2^, the modification with Lys48-linked ubiquitin chain takes place more efficiently on XPC bound to internucleosomal DNA, whereas XPC molecules on core particles are less prone to ubiquitination (Fei et al., 2011). The role of CRL4^DDB2^ in this context was confirmed by the following experimental manipulations: (i) depletion of either DDB2 or CUL4A using RNA interference, (ii) depletion of the nuclear ubiquitin pool by using the proteasome inhibitor MG132, or (iii) suppression of the ubiquitin pathway using a small-molecule E1 inhibitor. Alternatively, the ubiquitination of XPC was inhibited in mouse cells expressing a temperature-sensitive E1 mutant or with an XPC-green fluorescent fusion protein that makes the XPC protein refractory to ubiquitination. After each of these experimental manipulations, the XPC molecules were devoid of ubiquitin moieties and, as a consequence, almost completely relocated to nucleosome core particles (Fei et al., 2011). These findings demonstrate that one of the functions of CRL4^DDB2^-mediated ubiquitination is to retain XPC molecules at internucleosomal sites, which constitute DNA repair hotspots for the effective recruitment of TFIIH and further downstream NER factors (**Figure 2**). In the absence of CRL4^DDB2^ activity, more XPC binds to CPDs located in nucleosome core particles representing a less permissive chromatin environment with poor recruitment of downstream GG-NER factors. We concluded that the CRL4^DDB2^-mediated ubiquitination serves to establish a distinctive spatiotemporal distribution of the XPC sensor during the UV damage response, in particular to optimize the recruitment of NER factors in mammalian chromatin.

### Ubiquitin-dependent Extraction of DDB2 and XPC from Chromatin

**FIGURE 3:**
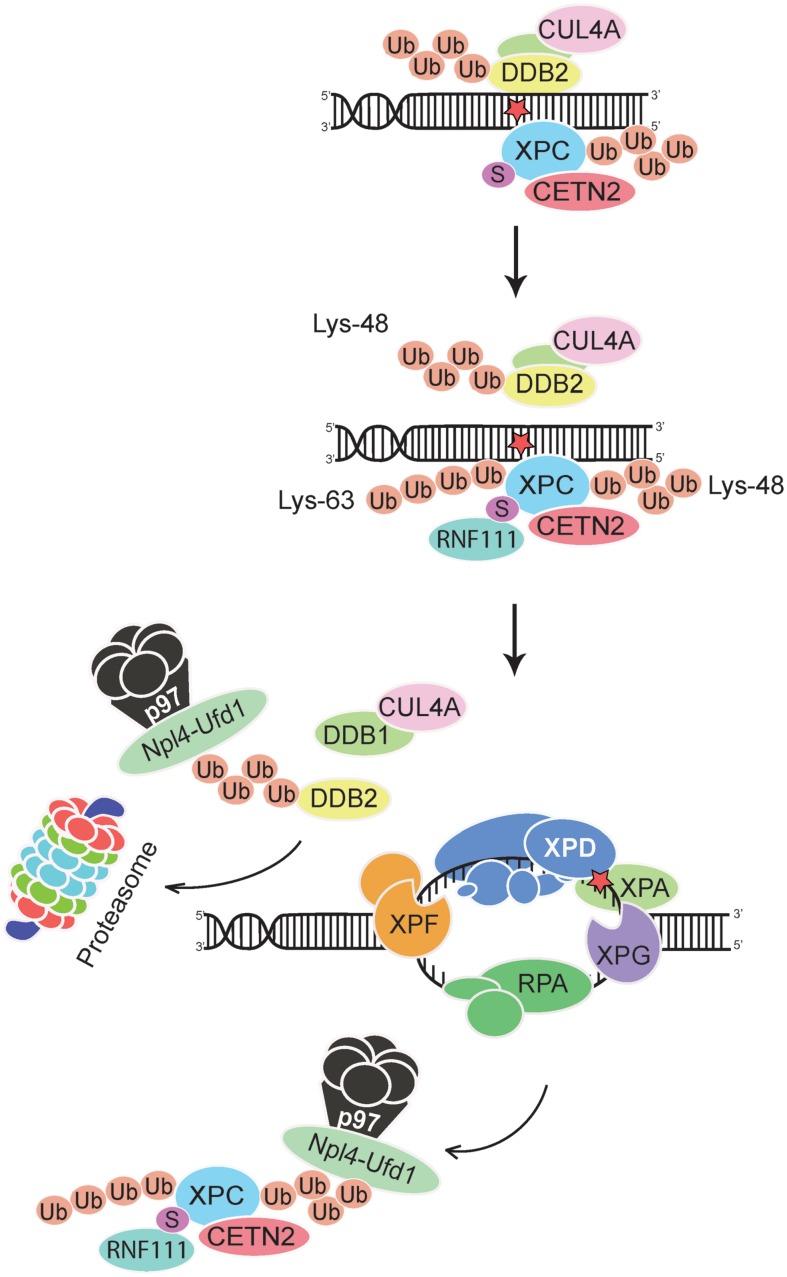
**Extraction of DDB2 and XPC from chromatin.** The p97 segregase coordinates GG-NER activity by removing Lys48-ubiquitinated DDB2 and Lys48-ubiquitinated XPC from chromatin, thus promoting downstream recognition (by the XPD subunit of TFIIH in conjunction with XPA and RPA) and double DNA incision. The XPC subunit is thought to leave the preincision complex after recruitment of TFIIH but before engagement of the DNA endonucleases XPF-ERCC1 and XPG (Scharer, 2013; van Cuijk et al., 2015). Ubiquitinated DDB2 is forwarded to the proteasome for degradation, whereas XPC is recycled by de-ubiquitination (He et al., 2014; Lubin et al., 2014; Puumalainen et al., 2014). Lys63-linked ubiquitin chains on XPC may further enhance these dynamic relocations at UV lesions by favoring the dissociation of DDB2 from XPC. See text for further details on the postulated dual role of CRL4^DDB2^ (generating Lys48-linked ubiquitin chains) and RNF111 (generating Lys63-linked ubiquitin chains) in regulating GG-NER activity. Npl4-Ufd1, adaptor complex that confers specificity to the p97 segregase; the UV radiation damage is symbolized by a red star.

Although the DDB2 damage detector is required for efficient recognition and excision of CPDs, Lys48-linked ubiquitin moieties elicit its proteolytic breakdown within few hours after exposure to UV light (Nag et al., 2001; Rapic-Otrin et al., 2002). This precipitous self-ubiquitination and degradation of DDB2 provides a time switch that limits the CRL4^DDB2^ ubiquitin ligase activity, and its regulatory effect on the XPC partner, to a short period after acute UV pulses. Due to DDB2 degradation, the proportion of ubiquitinated XPC diminishes progressively and, therefore, XPC can relocate from internucleosomal DNA segments to not yet processed residual UV lesions, essentially CPDs, located within the less amenable nucleosome core particles (Fei et al., 2011). These dynamic chromatin transitions, involving degradation of DDB2 and relocation of XPC, are triggered by the ubiquitin-selective p97 segregase, also known as VCP, (Puumalainen et al., 2014). Hexameric assemblies of p97 subunits convert ATP hydrolysis into mechanical activity to liberate ubiquitinated proteins from diverse subcellular substrates (Rouiller et al., 2000; Zhang et al., 2000). That p97 hexamers recognize ubiquitinated DDB2 and XPC was first demonstrated *in situ* on UV lesions spots in the nuclei of human cells. Second, it was confirmed biochemically that Lys48-ubiquitinated DDB2, XPC, and p97 are found in the same multi-protein complex (Puumalainen et al., 2014). This p97 recruitment to ubiquitinated DDB2 and XPC depends on adapter proteins (Meyer et al., 2000; Hänzelmann et al., 2011) known to confer substrate specificity to the p97 segregase (**Figure 3**).

Next, the p97 function was down regulated by RNA interference or, alternatively, by expression of a dominant-negative mutant (Ye et al., 2003) that still displays substrate-binding but is unable to exert segregase activity and, therefore, remains trapped on ubiquitinated proteins. The consequence of this diminished p97 activity is an enrichment of DDB2 and XPC in UV lesion spots, thus reflecting an excessive accumulation of these factors in damaged chromatin. The down-regulation of p97 inhibited the UV-induced proteolytic clearance of DDB2 and also increased the level of ubiquitinated XPC. However, despite their roles in the initiation of GG-NER activity, this induced persistence of DDB2 and XPC impaired UV lesion excision. Moreover, the compromised DNA repair efficiency resulting from p97 down regulation caused hypersensitivity to UV light and enhanced chromosomal aberrations after UV exposure.

The genome instability observed in UV-irradiated cells after p97 depletion was reversed by concurrent down-regulation of DDB2 or XPC (Puumalainen et al., 2014). These findings suggested that the uncontrolled accumulation of DDB2 or XPC is detrimental and that a tight regulation of their levels in chromatin is essential for genome stability. Elaborating on this hypothesis, one would expect that an excessive presence of one of these factors should be sufficient to destabilize the genome. In support of this hypothesis, it was found that under conditions of normal p97 activity, overexpression of wild-type DDB2 but not overexpression of a DNA-binding mutant, compromised UV lesion excision and increased the frequency of chromosomal aberrations following UV irradiation. Importantly, double overexpression experiments generating abnormally high levels of both DDB2 and p97 confirmed the expectation that the negative effects of DDB2 overexpression are reversed by concomitantly increasing p97 levels. Thus, a surplus of DDB2 enhances chromosomal aberrations only as long as its chromatin level exceeds the turnover capacity of the p97 segregase. Taken together, these findings point out that a strict spatial and temporal regulation of the chromatin homeostasis of DDB2 and its XPC partner by the p97 segregase is crucial for GG-NER activity (**Figure 3**).

## Conclusion

The XPC complex provides the generic initiator of GG-NER activity on the basis of its ability to sense the damage-dependent disruption of base pairs in double-stranded DNA and recruit the XPD scanner for bulky lesion recognition. An intriguing peculiarity of the XPC complex is that its function in initiating the excision of UV lesions is tightly regulated by NEDD8, sumo and ubiquitin modifiers. This special regulation is apparently not needed for the recognition and excision of other bulky lesions induced by chemical carcinogens or endogenous metabolic byproducts. An evolutionary perspective may help to understand the unique need for polypeptide modifier-dependent regulation of GG-NER activity in response to UV irradiation.

Evolution of life on our planet would have failed without the emergence of an effective DNA repair function dealing with UV lesions. Indeed, a vast majority of living organisms exposed to sunlight display rapid, efficient and secure molecular tools for the repair of UV lesions consisting of DNA photolyases. By visible light-driven catalysis, these DNA photolyases revert pyrimidine dimers (CPDs and 6-4PPs) to pyrimidine monomers without excision of bases, nucleoside or nucleotide residues (Sancar, 2003; Weber, 2005). Unlike other animals, however, placental mammals are devoid of this light-dependent DNA repair reaction, possibly because they originated from nocturnal ancestors (Essen and Klar, 2006). While returning to a diurnal life under sunlight, placental mammals were left with the GG-NER pathway (also known as “dark repair”) as the only means to process UV lesions in the exposed skin. In principle, many potential problems arise with this upgrade of GG-NER activity as the unique DNA repair defense against UV lesions. First, CPDs would escape repair because the XPC initiator is not able to detect this prevalent type of UV lesion. Second, once exposed to sunlight, skin cells would be faced with the simultaneous and uncontrolled cleavage of their genomic DNA at thousands or more chromosomal sites, which constitutes a striking threat to genome stability. Third, CPDs are formed evenly across the genomic DNA, including compacted chromatin sites that are poorly amenable to the GG-NER machinery.

The present review highlights NEDD8-, sumo- and ubiquitin-dependent mechanisms by which these problems related to “dark repair” by the GG-NER machinery are mitigated in human skin cells. First, the dedicated UV damage sensor DDB2 recruits its XPC partner to CPD lesions that, without DDB2, would remain undetected. Second, the GG-NER-initiating activity of XPC undergoes a tight spatial regulation. By recruitment of the CRL4^DDB2^ ligase responsible for XPC ubiquitination, the GG-NER reaction is in the beginning directed to highly amenable internucleosomal DNA segments that are accessible to downstream excision factors, thus protecting more compacted chromatin sites from premature incisions that might favor the fragmentation of chromosomes. Third, the repair-initiating activity of XPC undergoes a tight temporal regulation. By means of proteolytic breakdown triggered by the CRL4^DDB2^ ubiquitin ligase, the repair-stimulating action of DDB2 is self-limiting after an acute pulse of UV damage. Fourth, the physical interaction between DDB2 and XPC is counter-regulated by sumo and, presumably, the sumo-dependent RNF111 ubiquitin ligase. It is still an enigma how DDB2 and the XPC complex take advantage of histone-modifying enzymes as well as chromatin remodelers to relax chromatin regions and initiate the repair of compacted DNA substrates in a coordinated manner. It has, however, become clear that p97-mediated extraction of a surplus of ubiquitinated DDB2 and XPC is necessary to achieve optimal GG-NER activity and avoid molecular collisions with concomitant nuclear processes like transcription or DNA replication. Through addition of these NEDD8-, sumo- and ubiquitin-dependent control circuits, it has become possible during mammalian evolution to upgrade the GG-NER system as the only available DNA repair reaction protecting from UV-induced skin mutagenesis and carcinogenesis.

## Author Contributions

PR, CBP, and HN wrote the manuscript. PR and CP prepared the figures.

## Conflict of Interest Statement

The authors declare that the research was conducted in the absence of any commercial or financial relationships that could be construed as a potential conflict of interest.

## Abbreviations

6-4PP: (6-4) pyrimidine–pyrimidone photoproduct
BHD: β-Hairpin domain
CETN2: centrin 2
CPD: cyclobutane pyrimidine dimer
CUL4A: cullin 4A
DDB: damaged DNA-binding
ERCC1: excision repair cross-complementing 1
GG-NER: global-genome nucleotide excision repair
MPG: methylpurine-DNA glycosylase
NER: nucleotide excision repair
NEDD8: neural precursor cell expressed developmentally down-regulated 8
Npl4: nuclear protein localization 4 homolog
Oct4: octamer binding transcription factor 4
OGG1: 8-Oxo-guanine-DNA glycosylase
OTUD4: OTU deubiquitinase 4
RAD23B: human homolog of RAD23, B
RNF111: RING finger protein 111
RPA: replication protein A
ROC1: regulator of cullins 1
RPS27A: ubiquitin-40S ribosomal protein S27A
SMUG1: single strand-selective monofunctional uracil-DNA glycosylase 1
Sox2: sex determining region Y (SRY)-box 2
Sumo: small ubiquitin-related modifier
TC-NER: transcription-coupled nucleotide excision repair
TDG: thymine-DNA glycosylase
TFIIH: transcription factor IIH
TG: transglutaminase-like
UBA52: ubiquitin A-52
UBB: ubiquitin-B
UBC: ubiquitin-C
USP7: ubiquitin-specific processing protease 7
Ufd1: ubiquitin fusion degradation 1
UV: ultraviolet
VCP: valosin-containing protein
XP: xeroderma pigmentosum

## References

[B1] Akita M. Tak Y. S. Shimura T. Matsumoto S. Okuda-Shimizu Y. Shimizu Y. (2015). SUMOylation of xeroderma pigmentosum group C protein regulates DNA damage recognition during nucleotide excision repair. *Sci. Rep.* 5:10984 10.1038/srep10984 PMC4455304 26042670

[B2] Araki M. Masutani C. Takemura M. Uchida A. Sugasawa K. Kondoh J. (2001). Centrosome protein centrin 2/Caltractin 1 is part of the xeroderma pigmentosum group C complex that initiates global genome nucleotide excision repair. *J. Biol. Chem.* 276 18665–18672. 10.1074/jbc.m100855200 11279143

[B3] Araujo S. J. Tirode F. Coin F. Pospiech H. Syvaoja J. E. Stucki M. (2000). Nucleotide excision repair of DNA with recombinant human proteins: definition of the minimal set of factors, active forms of TFIIH, and modulation by CAK. *Genes Dev.* 14 349–359. 10673506 PMC316364

[B4] Bergink S. Toussaint W. Luijsterburg M. S. Dinant C. Alekseev S. Hoeijmakers J. H. J. (2012). Recognition of DNA damage by XPC coincides with disruption of the XPC-RAD23 complex. *J. Cell Biol.* 196 681–688. 10.1083/jcb.201107050 22431748 PMC3308700

[B5] Bernardes de Jesus B. M. Bjoras M. Coin F. Egly J. M. (2008). Dissection of the molecular defects caused by pathogenic mutations in the DNA repair factor XPC. *Mol. Cell. Biol.* 28 7225–7235. 10.1128/mcb.00781-08 18809580 PMC2593387

[B6] Bohr V. Smith C. A. Okumoto D. S. Hanawalt P. C. (1985). DNA repair in an active gene: removal of pyrimidine dimers from the DHFR gene of CHO cells is much more efficient than in the genome overall. *Cell* 40 359–369. 10.1016/0092-8674(85)901503 3838150

[B7] Brash D. E. (1988). UV mutagenic photoproducts in *Escherichia coli* and human cells: a molecular genetics perspective on human skin cancer. *Photochem. Photobiol.* 48 59–66. 10.1111/j.1751-1097.1988.tb02786.x 3064116

[B8] Brohée S. Helden J. (2006). Evaluation of clustering algorithsms for protein-protein interaction networks. *BMC Bioinformatics* 7:488 10.1186/1471-2105-7-488 PMC1637120 17087821

[B9] Brooks P. J. Wise D. S. Berry D. A. Kosmoski J. V. Smerdon M. J. Somers R. L. (2000). The oxidative DNA lesion 8,5’-(S)-cyclo-2’-deoxyadenosine is repaired by the nucleotide excision repair pathway and blocks gene expression in mammalian cells. *J. Biol. Chem.* 275 22355–22362. 10.1074/jbc.m002259200 10801836

[B10] Brueckner F. Hennecke U. Carell T. Cramer P. (2007). CPD damage recognition by transcribing RNA polymerase II. *Science* 315 859–862. 10.1126/science.1135400 17290000

[B11] Bunick C. G. Miller M. R. Fuller B. E. Fanning E. Chazin W. J. (2006). Biochemical and structural domain analysis of xeroderma pigmentosum complementation group C protein. *Biochemistry* 45 14965–14979. 10.1021/bi061370o 17154534 PMC2579963

[B12] Camenisch U. Träuflein D. Clement F. C. Fei J. Leitenstorfer A. Ferrando-May E. (2009). Two-stage dynamic DNA quality check by xeroderma pigmentosum group C protein. *EMBO J.* 28 2387–2399. 10.1038/emboj.2009.187 19609301 PMC2735174

[B13] Cattoglio C. Zhang E. T. Grubisic J. Chiba K. Fong Y. W. Tjian R. (2015). Functional and mechanistic studies of XPC DNA-repair complex as transcriptional coactivator in embryonic stem cells. *Proc. Natl. Acad. Sci. U.S.A.* 112 E2317–E2326. 10.1073/pnas.1505569112 25901318 PMC4426448

[B14] Cleaver J. E. Lam E. T. Revet I. (2009). Disorders of nucleotide excision repair: the genetic and molecular basis of heterogeneity. *Nat. Rev. Genet.* 10 756–768. 10.1038/nrg2663 19809470

[B15] Dantas T. J. Daly O. M. Conroy P. C. Tomas M. Wang Y. Lalor P. (2013). Calcium-binding capacity of centrin2 is required for linear POC5 assembly but not for nucleotide excision repair. *PLoS ONE* 8:e68487 10.1371/journal.pone.0068487 PMC3699651 23844208

[B16] Dantas T. J. Wang Y. Lalor P. Dockery P. Morrison C. G. (2011). Defective nucleotide excision repair with normal centrosome structures and functions in the absence of all vertebrate centrins. *J. Cell Biol.* 193 307–318. 10.1083/jcb.201012093 21482720 PMC3080269

[B17] D’Errico M. Lemma T. Calcagnile A. Santis L. P. D. Dogliotti E. (2007). Cell type and DNA damage specific response of human skin cells to environmental agents. *Mutat. Res.* 614 37–47. 10.1016/j.mrfmmm.2006.06.009 16879839

[B18] DiGiovanna J. J. Kraemer K. H. (2012). Shining a light on xeroderma pigmentosum. *J. Investig. Dermatol.* 132 785–796. 10.1038/jid.2011.426 22217736 PMC3279615

[B19] Donaldson M. R. Coldiron B. M. (2011). No end in sight: the skin cancer epidemic continues. *Semin. Cutan. Med. Surg.* 30 3–5. 10.1016/j.sder.2011.01.002 21540015

[B20] Essen L. O. Klar T. (2006). Light-driven DNA repair by photolyases. *Cell Mol. Life. Sci.* 63 1266–1277. 10.1007/s00018-005-5447-y 16699813 PMC11136382

[B21] Evans E. Fellows J. Coffer A. Wood R. D. (1997). Open complex formation around a lesion during nucleotide excision repair provides a structure for cleavage by human XPG protein. *EMBO J.* 16 625–638. 10.1093/emboj/16.3.625 9034344 PMC1169665

[B22] Fei J. Kaczmarek N. Luch A. Glas A. Carell T. Naegeli H. (2011). Regulation of nucleotide excision repair by UV-DDB: prioritization of damage recognition to internucleosomal DNA. *PLoS Biol.* 9:e1001183 10.1371/journal.pbio.1001183 PMC3201922 22039351

[B23] Fischer E. S. Scrima A. Böhm K. Matsumoto S. Lingaraju G. M. Faty M. (2011). The molecular basis of CRL4DDB2/CSA ubiquitin ligase architecture, targeting, and activation. *Cell* 147 1024–1039. 10.1016/j.cell.2011.10.035 22118460

[B24] Fitch M. E. Nakajima S. Yasui A. Ford J. M. (2003). In vivo recruitment of XPC to UV-induced cyclobutane pyrimidine dimers by the DDB2 gene product. *J. Biol. Chem.* 278 46906–46910. 10.1074/jbc.m307254200 12944386

[B25] Fong Y. W. Inouye C. Yamaguchi T. Cattoglio C. Grubisic I. Tjian R. (2011). A DNA repair complex functions as an Oct4/Sox2 coactivator in embryonic stem cells. *Cell* 147 120–131. 10.1016/j.cell.2011.08.038 21962512 PMC3216680

[B26] Gale J. M. Nissen K. A. Smerdon M. J. (1987). UV-induced formation of pyrimidine dimers in nucleosome core DNA is strongly modulated with a period of 10.3 bases. *Proc. Natl. Acad. Sci. U.S.A.* 84 6644–6648. 10.1073/pnas.84.19.6644 3477794 PMC299139

[B27] Gale J. M. Smerdon M. J. (1990). UV-induced (6-4) photoprducts are distributed differently than cyclobutane dimers in nucleosomes. *Photochem. Photobiol.* 51 411–417. 10.1111/j.1751-1097.1990.tb01732.x 2160660

[B28] Garinis G. A. Mitchell J. R. Moorhouse M. J. Hanada K. de Waard H. Vandeputte D. (2005). Transcriptome analysis reveals cyclobutane pyrimidine dimers as a major source of UV-induced DNA breaks. *EMBO J.* 24 3952–3962. 10.1038/sj.emboj.7600849 16252008 PMC1283948

[B29] Groisman R. Polanowska J. Kuraoka I. Sawada J.-I. Saijo M. Drapkin R. (2003). The ubiquitin ligase activity in the DDB2 and CSA complexes is differentially regulated by the COP9 signalosome in response to DNA damage. *Cell* 113 357–367. 10.1016/s0092-8674(03)00316-7 12732143

[B30] Guerrero-Santoro J. Kapetanaki M. G. Hsieh C. L. Gorbachinsky I. Levine A. S. Rapic-Otrin V. (2008). The Cullin 4B-based UV-damaged DNA-binding protein ligase binds to UV-damaged chromatin and ubiquitinates histone H2A. *Cancer Res.* 68 5014–5022. 10.1158/0008-5472.can-07-6162 18593899

[B31] Hanawalt P. C. Spivak G. (2008). Transcription-coupled DNA repair: two decades of progress and surprises. *Nat. Rev. Mol. Cell. Biol.* 9 958–970. 10.1038/nrm2549 19023283

[B32] Hänzelmann P. Buchberger A. Schindelin H. (2011). Hierarchical binding of cofactors to the AAA ATPase p97. *Structure* 19 833–843. 10.1016/j.str.2011.03.018 21645854

[B33] He J. Zhu Q. Wani G. Sharma N. Han C. Qian J. (2014). Ubiquitin-specific protease 7 regulates nucleotide excision repair through deubiquitinating XPC protein and preventing XPC protein from undergoing ultraviolet light-induced and VCP/p97 protein-regulated proteolysis. *J. Biol. Chem.* 289 27278–27289. 10.1074/jbc.m114.589812 25118285 PMC4175359

[B34] Hey T. Lipps G. Sugasawa K. Iwai S. Hanaoka F. Krauss G. (2002). The XPC-HR23B complex displays high affinity and specificity for damaged DNA in a true-equilibrium fluorescence assay. *Biochemistry* 41 6583–6587. 10.1021/bi012202t 12022861

[B35] Hollander M. C. Philburn R. T. Patterson A. D. Velasco-Miguel S. Friedberg E. C. Linnoila R. I. (2005). Deletion of XPC leads to lung tumors in mice and is associated with early events in human lung carcinogenesis. *Proc. Natl. Acad. Sci. U.S.A.* 102 13200–13205. 10.1073/pnas.0503133102 16141330 PMC1201581

[B36] Huang J. C. Svoboda D. L. Reardon J. T. Sancar A. (1992). Human nucleotide excision nuclease removes thymine dimers from DNA by incising the 22nd phosphodiester bond 5’ and the 6th phosphodiester bond 3’ to the photodimer. *Proc. Natl. Acad. Sci. U.S.A.* 89 3664–3668. 10.1073/pnas.89.8.3664 1314396 PMC48929

[B37] Hwang B. J. Ford J. M. Hanawalt P. C. Chu G. (1999). Expression of the p48 xeroderma pigmentosum gene is p53-dependent and is involved in global genomic repair. *Proc. Natl. Acad. Sci. U.S.A.* 96 424–428. 10.1073/pnas.96.2.424 9892649 PMC15152

[B38] Itoh T. Cado D. Kamide R. Linn S. (2004). DDB2 gene disruption leads to skin tumors and resistance to apoptosis after exposure to ultraviolet light but not a chemical carcinogen. *Proc. Natl. Acad. Sci. U.S.A.* 101 2052–2057. 10.1073/pnas.0306551101 14769931 PMC357050

[B39] Jing Y. Taylor J. S. Kao J. F. L. (1998). Thermodynamic and base-pairing studies of matched and mismatched DNA dodecamer duplexes containing cis-syn, (6-4) and Dewar photoproducts of TT. *Nucleic Acids Res.* 26 3845–3853. 10.1093/nar/26.16.3845 9685504 PMC147757

[B40] Kapetanaki M. G. Guerrero-Santoro J. Bisi D. C. Hsieh C. L. Rapic-Otrin V. Levine A. S. (2006). The DDB1-CUL4ADDB2 ubiquitin ligase is deficient in xeroderma pigmentosum group E and targets histone H2A at UV-damaged DNA sites. *Proc. Natl. Acad. Sci. U.S.A.* 103 2588–2593. 10.1073/pnas.0511160103 16473935 PMC1413840

[B41] Khorasanizadeh S. (2004). The nucleosome. *Cell* 116 259–272. 10.1016/s0092-8674(04)00044-3 14744436

[B42] Kim J. K. Soni S. D. Arakali A. V. Wallace J. C. Alderfer J. L. (1995). Solution structure of a nucleic acid photoproduct of deoxyfluorouridylyl-(3’-5’)-thymidine monophosphate (d-FpT) determined by NMR and restrained molecular dynamics: structural comparison of two sequence isomer photoadducts (d-U5p5T and d-T5p5U). *Nucleic Acids Res.* 23 1810–1815. 10.1093/nar/23.10.1810 7784187 PMC306940

[B43] Knox R. J. Lydall D. A. Friedlos F. Basham C. Roberts J. J. (1987). The effect of monofunctional or difunctional platinum adducts and of various other associated DNA damage on the expression of transfected DNA in mammalian cell lines sensitive or resistant to difunctional agents. *Biochim. Biophys. Acta* 908 214–223. 10.1016/0167-4781(87)90101-1 3567197

[B44] Krasikova Y. S. Rechkunova N. I. Maltseva E. A. Craescu C. T. Petruseva I. O. Lavrik O. I. (2012). Influence of centrin 2 on the interaction of nucleotide excision repair factors with damaged DNA. *Biochemistry (Moscow)* 77 346–353. 10.1134/s0006297912040050 22809153

[B45] Kulaksiz G. Reardon J. T. Sancar A. (2005). Xeroderma pigmentosum complementation group E protein (XPE/DDB2): purification of various complexes of XPE and analyses of their damaged DNA binding and putative DNA repair properties. *Mol. Cell. Biol.* 25 9784–9792. 10.1128/mcb.25.22.9784-9792.2005 16260596 PMC1280284

[B46] Kuraoka I. Bender C. Romieu A. Cadet J. Wood R. D. Lindahl T. (2000). Removal of oxygen free-radical-induced 5’,8-purine cyclodeoxynucleosides from DNA by the nucleotide excision-repair pathway in human cells. *Proc. Natl. Acad. Sci. U.S.A.* 97 3832–3837. 10.1073/pnas.070471597 10759556 PMC18102

[B47] Li C. L. Golebiowski F. M. Onishi Y. Samara N. L. Sugasawa K. Yang W. (2015). Tripartite DNA lesion recognition and verification by XPC, TFIIH, and XPA in nucleotide excision repair. *Mol. Cell.* 59 1025–1034. 10.1016/j.molcel.2015.08.012 26384665 PMC4617536

[B48] Liu L. Lee S. Zhang J. Peters S. B. Hannah J. Zhang Y. (2009). CUL4A abrogation augments DNA damage response and protection against skin carcinogenesis. *Mol. Cell.* 34 451–460. 10.1016/j.molcel.2009.04.020 19481525 PMC2722740

[B49] Lopes M. Foiani M. Sogo J. M. (2006). Multiple mechanisms control chromosome integrity after replication fork uncoupling and restart at irreparable UV lesions. *Mol. Cell.* 21 15–27. 10.1016/j.molcel.2005.11.015 16387650

[B50] Lubin A. Zhang L. Chen H. White V. M. Gong F. (2014). A human XPC protein interactome–a resource. *Int. J. Mol. Sci.* 15 141–158. 10.3390/ijms15010141 24366067 PMC3907802

[B51] Marteijn J. A. Lans H. Vermeulen W. Hoeijmakers J. H. J. (2014). Understanding nucleotide excision repair and its roles in cancer and ageing. *Nat. Rev. Mol. Cell Biol.* 15 465–481. 10.1038/nrm3822 24954209

[B52] Masutani C. Kusumoto R. Yamada A. Dohmae N. Yokoi M. Yuasa M. (1999). The XPV (xeroderma pigmentosum variant) gene encodes human DNA polymerase eta. *Nature* 399 700–704. 10.1038/21447 10385124

[B53] Mathieu N. Kaczmarek N. Naegeli H. (2010). Strand- and site-specific DNA lesion demarcation by the xeroderma pigmentosum group D helicase. *Proc. Natl. Acad. Sci. U.S.A.* 107 17545–17550. 10.1073/pnas.1004339107 20876134 PMC2955138

[B54] Matsumoto S. Fischer E. S. Yasuda T. Dohmae N. Iwai S. Mori T. (2015). Functional regulation of the DNA damage-recognition factor DDB2 by ubiquitination and interaction with xeroderma pigmentosum group C protein. *Nucleic Acids Res.* 43 1700–1713. 10.1093/nar/gkv038 25628365 PMC4330392

[B55] McAteer K. Jing Y. Kao J. Taylor J. S. Kennedy M. A. (1998). Solution-state structure of a DNA dodecamer duplex containing a Cis-Syn thymine cyclobutane dimer, the major UV photoproduct of DNA. *J. Mol. Biol.* 282 1013–1032. 10.1006/jmbi.1998.2062 9753551

[B56] Melis J. P. M. Luijten M. Mullenders L. H. F. van Steeg H. (2011). The role of XPC: implications in cancer and oxidative DNA damage. *Mutat. Res.* 728 107–117. 10.1016/j.mrrev.2011.07.001 21763452 PMC3203325

[B57] Meyer H. H. Shorter J. G. Seemann J. Pappin D. Warren G. (2000). A complex of mammalian Ufd1 and Npl4 links the AAA-ATPase, p97, to ubiquitin and nuclear transport pathways. *EMBO J.* 19 2181–2192. 10.1093/emboj/19.10.2181 10811609 PMC384367

[B58] Miao F. Bouziane M. Dammann R. Masutani C. Hanaoka F. Pfeifer G. (2000). 3-Methyladenine-DNA glycosylase (MPG protein) interacts with human RAD23 proteins. *J. Biol. Chem.* 275 28433–28438. 10.1074/jbc.m001064200 10854423

[B59] Min J.-H. Pavletich N. P. (2007). Recognition of DNA damage by the Rad4 nucleotide excision repair protein. *Nature* 449 570–575. 10.1038/nature06155 17882165

[B60] Missura M. Buterin T. Hindges R. Hübscher U. Kaspárková J. Brabec V. (2001). Double-check probing of DNA bending and unwinding by XPA-RPA: an architectural function in DNA repair. *EMBO J.* 20 3554–3564. 10.1093/emboj/20.13.3554 11432842 PMC125508

[B61] Mitchell D. L. Cleaver J. E. Epstein J. H. (1990). Repair of pyrimidine (6-4)pyrimidone photoproducts in mouse skin. *J. Invest. Dermatol.* 95 55–59. 10.1111/1523-1747.ep12873312 2366001

[B62] Moggs J. G. Yarema K. J. Essigmann J. M. Wood R. D. (1996). Analysis of incision sites produced by human cell extracts and purified proteins during nucleotide excision repair of a 1,3-intrastrand d(GpTpG)-cisplatin adduct. *J. Biol. Chem.* 271 7177–7186. 10.1074/jbc.271.12.7177 8636155

[B63] Moser J. Kool H. Giakzidis I. Caldecott K. Mullenders L. H. F. Fousteri M. I. (2007). sealing of chromosomal DNA nicks during nucleotide excision repair requires XRCC1 and DNA ligase IIIα in a cell-cycle-specific manner. *Mol. Cell.* 27 311–323. 10.1016/j.molcel.2007.06.014 17643379

[B64] Moser J. Volker M. Kool H. Alekseev S. Vrieling H. Yasui A. (2005). The UV-damaged DNA binding protein mediates efficient targeting of the nucleotide excision repair complex to UV-induced photo lesions. *DNA Rep.* 4 571–582. 10.1016/j.dnarep.2005.01.001 15811629

[B65] Mu H. Geacintov N. E. Zhang Y. Broyde S. (2015). Recognition of damaged DNA for nucleotide excision repair: a correlated motion mechanism with a mismatched cis-syn thymine dimer lesion. *Biochemistry* 54 5263–5267. 10.1021/acs.biochem.5b00840 26270861 PMC4748833

[B66] Nag A. Bondar T. Shiv S. Raychaudhuri P. (2001). The xeroderma pigmentosum group E gene product DDB2 is a specific target of Cullin 4A in mammalian cells. *Mol. Cell. Biol.* 21 6738–6747. 10.1128/mcb.21.20.6738-6747.2001 11564859 PMC99852

[B67] Ng J. M. Y. Vermeulen W. ven der Horst G. T. Bergink S. Sugasawa K. Vrieling H. (2003). A novel regulation mechanism of DNA repair by damage-induced and RAD23-dependent stabilization of xeroderma pigmentosum group C protein. *Genes Dev.* 17 1630–1645. 10.1101/gad.260003 12815074 PMC196135

[B68] Nichols A. F. Itoh T. Graham J. A. Liu W. Yamaizumi M. Linn S. (2000). Human damage-specific DNA-binding protein p48. Characterization of XPE mutations and regulation following UV irradiation. *J. Biol. Chem.* 275 21422–21428. 10.1074/jbc.m000960200 10777490

[B69] Nishi R. Okuda Y. Watanabe E. Mori T. Iwai S. Masutani C. (2005). Centrin 2 stimulates nucleotide excision repair by interacting with xeroderma pigmentosum group C protein. *Mol. Cell. Biol.* 25 5664–5674. 10.1128/mcb.25.13.5664-5674.2005 15964821 PMC1156980

[B70] Ogi T. Limsirichaikul S. Overmeer R. M. Volker M. Takenaka K. Cloney R. (2010). Three DNA polymerases, recruited by different mechanisms, carry out NER repair synthesis in human cells. *Mol. Cell.* 37 714–727. 10.1016/j.molcel.2010.02.009 20227374

[B71] Osakabe A. Tachiwana H. Kagawa W. Horikoshi N. Matsumoto S. Hasegawa M. (2015). Structural basis of pyrimidine-pyrimidone (6-4) photoproduct recognition by UV-DDB in the nucleosome. *Sci. Rep.* 5:16330 10.1038/srep16330 PMC4648065 26573481

[B72] Poulsen S. L. Hansen R. K. Wagner S. A. van Cuijk L. van Belle G. J. Streicher W. (2013). RNF111/Arkadia is a SUMO-targeted ubiquitin ligase that facilitates the DNA damage response. *J. Cell Biol.* 201 797–807. 10.1083/jcb.201212075 23751493 PMC3678163

[B73] Puumalainen M.-R. Lessel D. Rüthemann P. Kaczmarek N. Bachmann K. Ramadan K. (2014). Chromatin retention of DNA damage sensors DDB2 and XPC through loss of p97 segregase causes genotoxicity. *Nat. Commun.* 5:3695 10.1038/ncomms4695 PMC4007632 24770583

[B74] Puumalainen M. R. Ruthemann P. Min J. H. Naegeli H. (2016). Xeroderma pigmentosum group C sensor: unprecedented recognition strategy and tight spatiotemporal regulation. *Cell Mol. Life. Sci.* 73 547–566. 10.1007/s00018-015-2075-z 26521083 PMC4713717

[B75] Rapic-Otrin V. McLenigan M. P. Bisi D. C. Gonzalez M. Levine A. S. (2002). Sequential binding of UV DNA damage binding factor and degradation of the p48 subunit as early events after UV irradiation. *Nucleic Acids Res.* 30 2588–2598. 10.1093/nar/30.11.2588 12034848 PMC117178

[B76] Reardon J. T. Nichols A. F. Keeney S. Smith C. A. Taylor J. S. Linn S. (1993). Comparative analysis of binding of human damaged DNA-binding protein (XPE) and *Escherichia coli* damage recognition protein (UvrA) to the major ultraviolet photoproducts: T[c,s]T, T[t,s]T, T[6-4]T, and T[Dewar]T. *J. Biol. Chem.* 268 21301–21308. 8407968

[B77] Reardon J. T. Sancar A. (2003). Recognition and repair of the cyclobutane thymine dimer, a major cause of skin cancers, by the human excision nuclease. *Genes Dev.* 17 2539–2551. 10.1101/gad.1131003 14522951 PMC218148

[B78] Rouiller I. Butel V. M. Latterich M. Milligan R. A. Wilson-Kubalek E. M. (2000). A major conformational change in p97 AAA ATPase upon ATP binding. *Mol. Cell.* 6 1485–1490. 10.1016/s1097-2765(00)00144-1 11163220

[B79] Sancar A. (2003). Structure and function of DNA photolyase and cryptochrome blue-light photoreceptors. *Chem. Rev.* 103 2203–2238. 10.1021/cr0204348 12797829

[B80] Scharer O. D. (2013). Nucleotide excision repair in eukaryotes. *Cold Spring Harb. Perspect. Biol.* 5:a012609 10.1101/cshperspect.a012609 PMC3783044 24086042

[B81] Schul W. Jans J. Rijksen Y. M. Klemann K. H. Eker A. P. de Wit J. (2002). Enhanced repair of cyclobutane pyrimidine dimers and improved UV resistance in photolyase transgenic mice. *EMBO J.* 21 4719–4729. 10.1093/emboj/cdf456 12198174 PMC125407

[B82] Scrima A. Koníčková R. Czyzewski B. K. Kawasaki Y. Jeffrey P. D. Groisman R. (2008). Structural basis of UV DNA-damage recognition by the DDB1–DDB2 complex. *Cell* 135 1213–1223. 10.1016/j.cell.2008.10.045 19109893 PMC2676164

[B83] Shimizu Y. Iwai S. Hanaoka F. Sugasawa K. (2003). Xeroderma pigmentosum group C protein interacts physically and functionally with thymine DNA glycosylase. *EMBO J.* 22 164–173. 10.1093/emboj/cdg016 12505994 PMC140069

[B84] Shimizu Y. Uchimura Y. Dohmae N. Saitho H. Hanaoka F. Sugasawa K. (2010). Stimulation of DNA glycosylase activities by XPC protein complex: roles of protein-protein interactions. *J. Nucleic Acids* 2010 805698 10.4061/2010/805698 PMC2925305 20798892

[B85] Staresincic L. Fagbemi A. F. Enzlin J. H. Gourdin A. M. Wijgers N. Dunand-Sauthier I. (2009). Coordination of dual incision and repair synthesis in human nucleotide excision repair. *EMBO J.* 28 1111–1120. 10.1038/emboj.2009.49 19279666 PMC2683701

[B86] Straub K. M. Meehan T. Burlingame A. L. Calvin M. (1977). Identification of the major adducts formed by reaction of benzo(a)pyrene diol epoxide with DNA in vitro. *Proc. Natl. Acad. Sci. U.S.A.* 74 5285–5289. 10.1073/pnas.74.12.5285 271953 PMC431688

[B87] Sugasawa K. Akagi J.-I. Nishi R. Iwai S. Hanaoka F. (2009). Two-step recognition of DNA damage for mammalian nucleotide excision repair: directional binding of the XPC complex and DNA strand scanning. *Mol. Cell* 36 642–653. 10.1016/j.molcel.2009.09.035 19941824

[B88] Sugasawa K. Masutani C. Uchida A. Maekawa T. van der Spek P. J. Bootsma D. (1996). HHR23B, a human Rad23 homolog, stimulates XPC protein in nucleotide excision repair in vitro. *Mol. Cell. Biol.* 16 4852–4861. 10.1128/MCB.16.9.4852 8756644 PMC231487

[B89] Sugasawa K. Ng J. M. Y. Masutani C. Iwai S. van der Spek P. J. Eker A. P. M. (1998). Xeroderma pigmentosum group C protein complex is the initiator of global genome nucleotide excision repair. *Mol. Cell.* 2 223–232. 10.1016/s1097-2765(00)80132-x 9734359

[B90] Sugasawa K. Okamoto T. Shimizu Y. Masutani C. Iwai S. Hanaoka F. (2001). A multistep damage recognition mechanism for global genomic nucleotide excision repair. *Genes Dev.* 15 507–521. 10.1101/gad.866301 11238373 PMC312644

[B91] Sugasawa K. Okuda Y. Saijo M. Nishi R. Matsuda N. Chu G. (2005). UV-Induced ubiquitylation of XPC protein mediated by UV-DDB-ubiquitin ligase complex. *Cell* 121 387–400. 10.1016/j.cell.2005.02.035 15882621

[B92] Tang J. Y. Hwang B. J. Ford J. M. Hanawalt P. C. Chu G. (2000). Xeroderma pigmentosum p48 gene enhances global genomic repair and suppresses UV-induced mutagenesis. *Mol. Cell.* 5 737–744. 10.1016/s1097-2765(00)80252-x 10882109 PMC2894271

[B93] Telford D. J. Stewart B. W. (1989). Characteristics of chromatin release during digestion of nuclei with micrococcal nuclease: preferential solubilization of nascent RNA at low enzyme concentration. *Intern. J. Biochem.* 21 1235–1240. 10.1016/0020-711x(89)90009-8 2482203

[B94] Thoma F. (2005). Repair of UV lesions in nucleosomes – intrinsic properties and remodeling. *DNA Rep.* 4 855–869. 10.1016/j.dnarep.2005.04.005 15925550

[B95] Trego K. S. Turchi J. J. (2006). Pre-steady-state binding of damaged DNA by XPC-hHR23B reveals a kinetic mechanism for damage discrimination. *Biochemistry* 45 1961–1969. 10.1021/bi051936t 16460043 PMC2435173

[B96] Uchida A. Sugasawa K. Masutani C. Dohmae N. Araki M. Yokoi M. (2002). The carboxy-terminal domain of the XPC protein plays a crucial role in nucleotide excision repair through interactions with transcription factor IIH. *DNA Rep.* 1 449–461. 10.1016/S1568-7864(02)00031-9 12509233

[B97] Usher-Smith J. A. Emery J. Kassianos A. P. Walter F. M. (2014). Risk prediction models for melanoma: a systematic review. *Cancer Epidemiol. Biomarkers. Prev.* 23 1450–1463. 10.1158/1055-9965.epi-14-0295 24895414

[B98] van Cuijk L. van Belle G. J. Turkyilmaz Y. Poulsen S. L. Janssens R. C. Theil A. F. (2015). SUMO and ubiquitin-dependent XPC exchange drives nucleotide excision repair. *Nat. Commun.* 6:7499 10.1038/ncomms8499 PMC4501428 26151477

[B99] Vermeulen W. Fousteri M. (2013). Mammalian transcription-coupled excision repair. *Cold Spring Harb. Perspect. Biol.* 5:a012625 10.1101/cshperspect.a012625 PMC3721277 23906714

[B100] Volker M. Moné M. J. Karmakar P. van Hoffen A. Schul W. Vermeulen W. (2001). Sequential assembly of the nucleotide excision repair factors in vivo. *Mol. Cell.* 8 213–224. 10.1016/s1097-2765(01)00281-7 11511374

[B101] Wakasugi M. Sancar A. (1998). Assembly, subunit composition, and footprint of human DNA repair excision nuclease. *Proc. Natl. Acad. Sci. U.S.A.* 95 6669–6674. 10.1073/pnas.95.12.6669 9618470 PMC22593

[B102] Wakasugi M. Shimizu M. Morioka H. Linn S. Nikaido O. Matsunaga T. (2001). Damaged DNA-binding protein DDB stimulates the excision of cyclobutane pyrimidine dimers in vitro in concert with XPA and replication protein A. *J. Biol. Chem.* 276 15434–15440. 10.1074/jbc.m011177200 11278856

[B103] Wang H. Zhai L. Xu J. Joo H.-Y. Jackson S. Erdjument-Bromage H. (2006). Histone H3 and H4 ubiquitylation by the CUL4-DDB-ROC1 ubiquitin ligase facilitates cellular response to DNA damage. *Mol. Cell.* 22 383–394. 10.1016/j.molcel.2006.03.035 16678110

[B104] Wang Q. E. Zhu Q. Wani G. El-Mahdy M. A. Li J. Wani A. A. (2005). DNA repair factor XPC is modified by SUMO-1 and ubiquitin following UV irradiation. *Nucleic Acids Res.* 33 4023–4034. 10.1093/nar/gki684 16030353 PMC1178000

[B105] Weber S. (2005). Light-driven enzymatic catalysis of DNA repair: a review of recent biophysical studies on photolyase. *Biochim. Biophys. Acta* 1707 1–23. 10.1016/j.bbabio.2004.02.010 15721603

[B106] Wittschieben B. O. Iwai S. Wood R. D. (2005). DDB1-DDB2 (xeroderma pigmentosum group E) protein complex recognizes a cyclobutane pyrimidine dimer, mismatches, apurinic/apyrimidinic sites, and compound lesions in DNA. *J. Biol. Chem.* 280 39982–39989. 10.1074/jbc.m507854200 16223728

[B107] Xie Z. Liu S. Zhang Y. Wang Z. (2004). Roles of Rad23 protein in yeast nucleotide excision repair. *Nucleic Acids Res.* 32 5981–5990. 10.1093/nar/gkh934 15545636 PMC534619

[B108] Yang A. Miron S. Mouawad L. Duchambon P. Blouquit Y. Craescu C. T. (2006). Flexibility and plasticity of human centrin 2 binding to the xeroderma pigmentosum group C protein (XPC) from nuclear excision repair. *Biochemistry* 45 3653–3663. 10.1021/bi0524868 16533048

[B109] Yasuda T. Sugasawa K. Shimizu Y. Iwai S. Shiomi T. Hanaoka F. (2005). Nucleosomal structure of undamaged DNA regions suppresses the non-specific DNA binding of the XPC complex. *DNA Rep.* 4 389–395. 10.1016/j.dnarep.2004.10.008 15661662

[B110] Ye Y. Meyer H. H. Rapoport T. A. (2003). Function of the p97-Ufd1-Npl4 complex in retrotranslocation from the ER to the cytosol: dual recognition of nonubiquitinated polypeptide segments and polyubiquitin chains. *J. Cell Biol.* 162 71–84. 10.1083/jcb.200302169 12847084 PMC2172719

[B111] Yeh J. I. Levine A. S. Du S. Chinte U. Ghodke H. Wang H. (2012). Damaged DNA induced UV-damaged DNA-binding protein (UV-DDB) dimerization and its roles in chromatinized DNA repair. *Proc. Natl. Acad. Sci. U.S.A.* 109 E2737–E2746. 10.1073/pnas.1110067109 22822215 PMC3478663

[B112] Yokoi M. Masutani C. Maekawa T. Sugasawa K. Ohkuma Y. Hanaoka F. (2000). The xeroderma pigmentosum group C protein complex XPC-HR23B plays an important role in the recruitment of transcription factor IIH to damaged DNA. *J. Biol. Chem.* 275 9870–9875. 10.1074/jbc.275.13.9870 10734143

[B113] Zhang E. T. He Y. Grog P. Fong Y. W. Nogales E. Tjian R. (2015). Architecture of the human XPC DNA repair and stem cell coactivator complex. *Proc. Natl. Acad. Sci. U.S.A.* 48 14817–14822. 10.1073/pnas.1520104112 26627236 PMC4672820

[B114] Zhang X. Shaw A. Bates P. A. Newman R. H. Gowen B. Orlova E. (2000). Structure of the AAA ATPase p97. *Mol. Cell.* 6 1473–1484. 10.1016/s1097-2765(00)00143-x 11163219

